# Supplementary material for: A robust zirconium amino acid metal-organic framework for proton conduction
Source: Nat Commun. 2018 Nov 22;9:4937. doi: 10.1038/s41467-018-07414-4 (PMC6250719; doi:10.1038/s41467-018-07414-4)
Supplement: Supplementary file 1 — Supplementary Information [file 41467_2018_7414_MOESM1_ESM.pdf]

Supplementary Information

A Robust Zirconium Amino Acid Metal-Organic Framework for Proton Conduction

Sujing Wang et al.

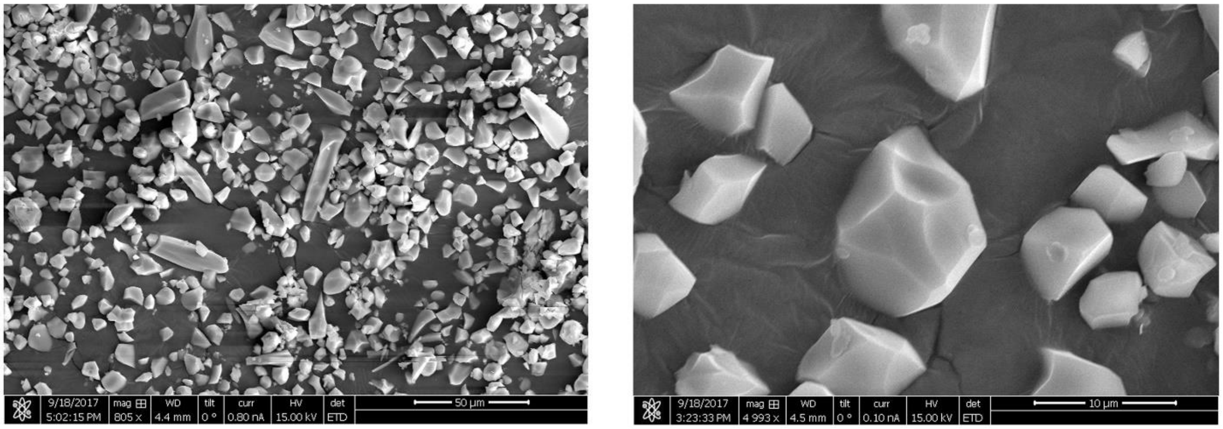

Supplementary Figure 1. SEM images of MIP-202(Zr) sample with different magnifications obtained from reaction without stirring.

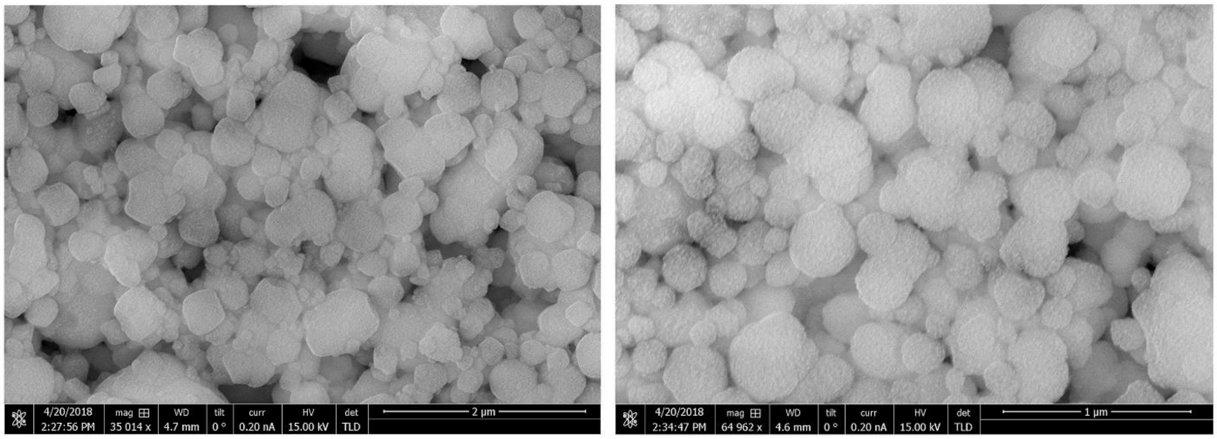

Supplementary Figure 2. SEM images of MIP-202(Zr) sample with different magnifications obtained from reaction with stirring.

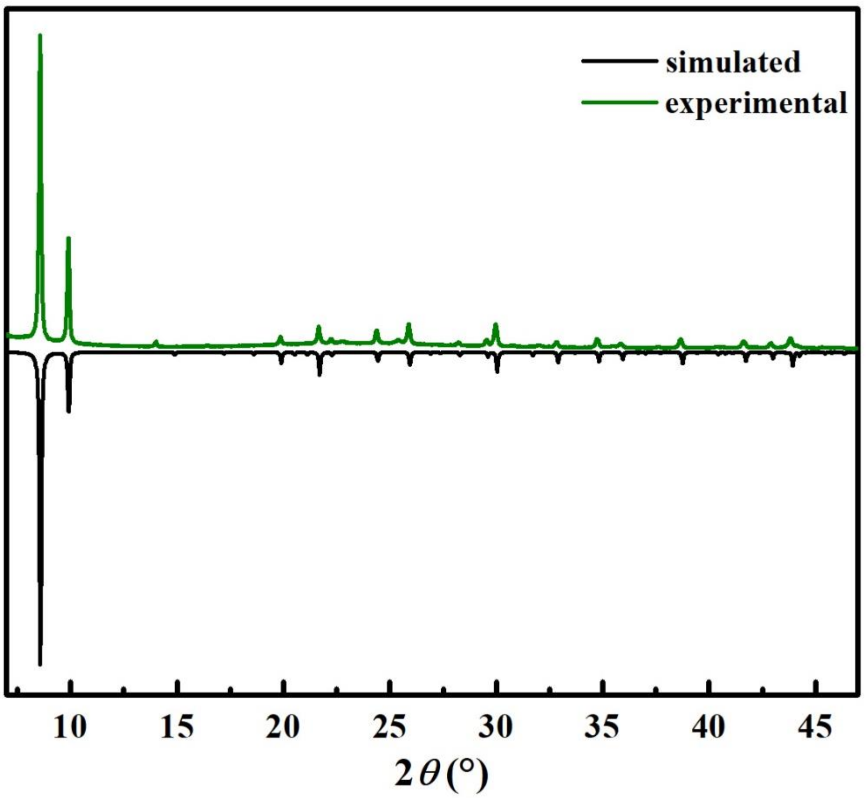

Supplementary Figure 3 Comparison between the experimental PXRD pattern and the calculated data obtained from the DFT optimized geometry of the MIP-202(Zr) solid.

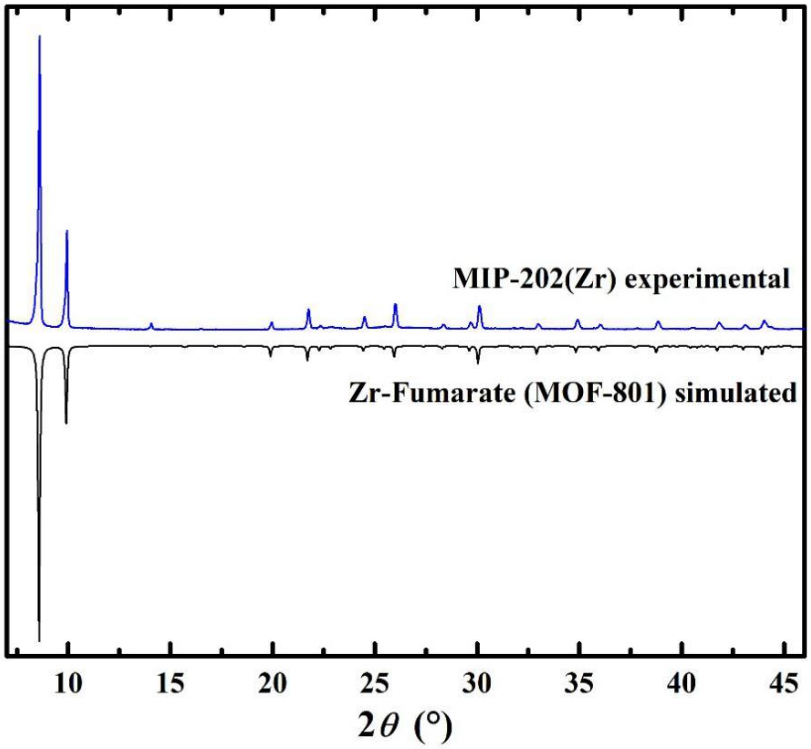

Supplementary Figure 4. PXRD pattern comparison of MIP-202(Zr) and Zr-Fumarate.

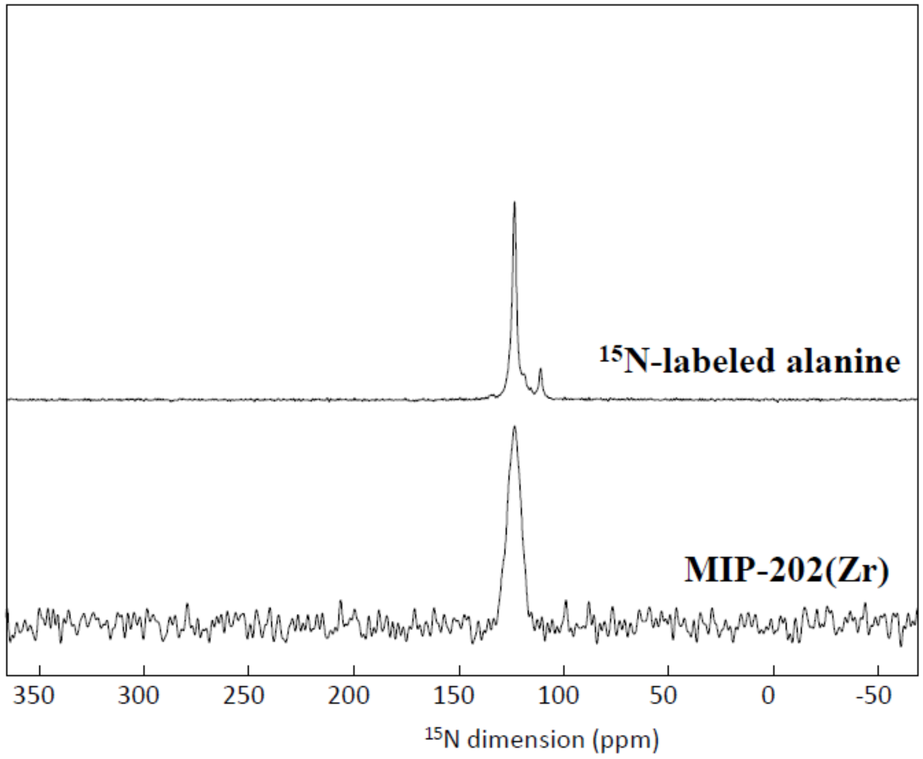

Supplementary Figure 5. ^{15}N CPMAS (11.7 T, MAS 10 kHz) solid-state NMR spectra of MIP-202(Zr) (^{15}N natural abundance aspartic linker) compared to that of zwitterionic alanine (^{15}N-^{13}C doubly-labeled compound).

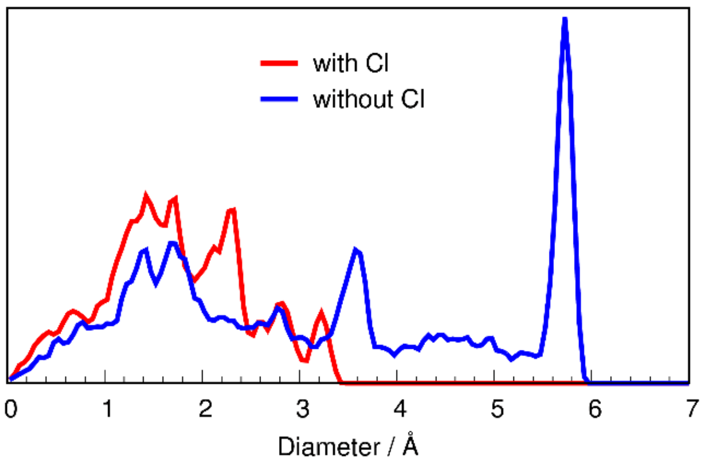

Supplementary Figure 6. Simulated Pore size distribution (PSD) of MIP-202(Zr) with and without the Cl^- ions present in the pores obtained by applying the geometric method to the crystal structure

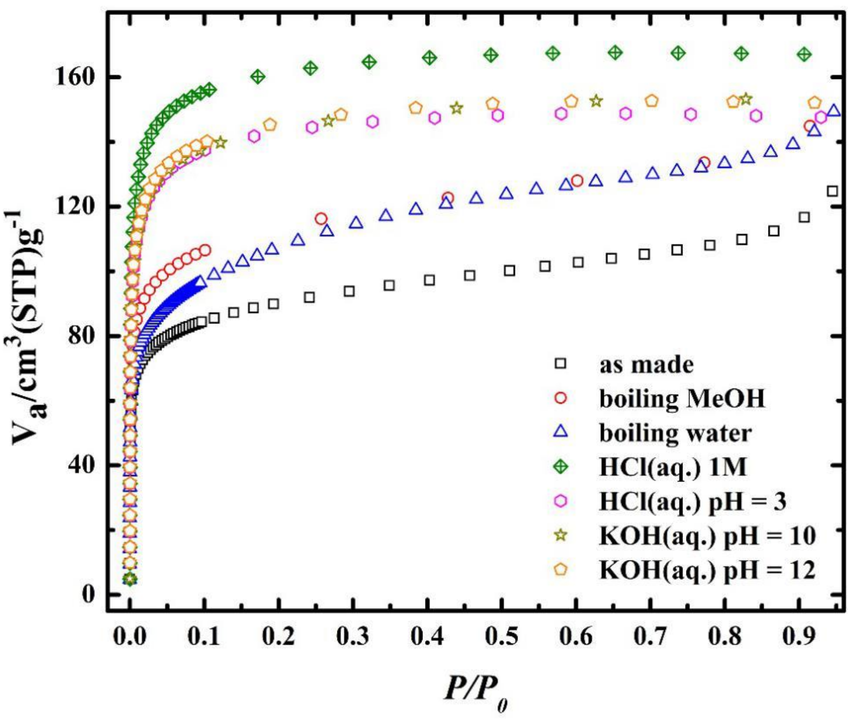

Supplementary Figure 7. Nitrogen adsorption isotherm comparison of MIP-202(Zr) samples before and after various chemical treatments.

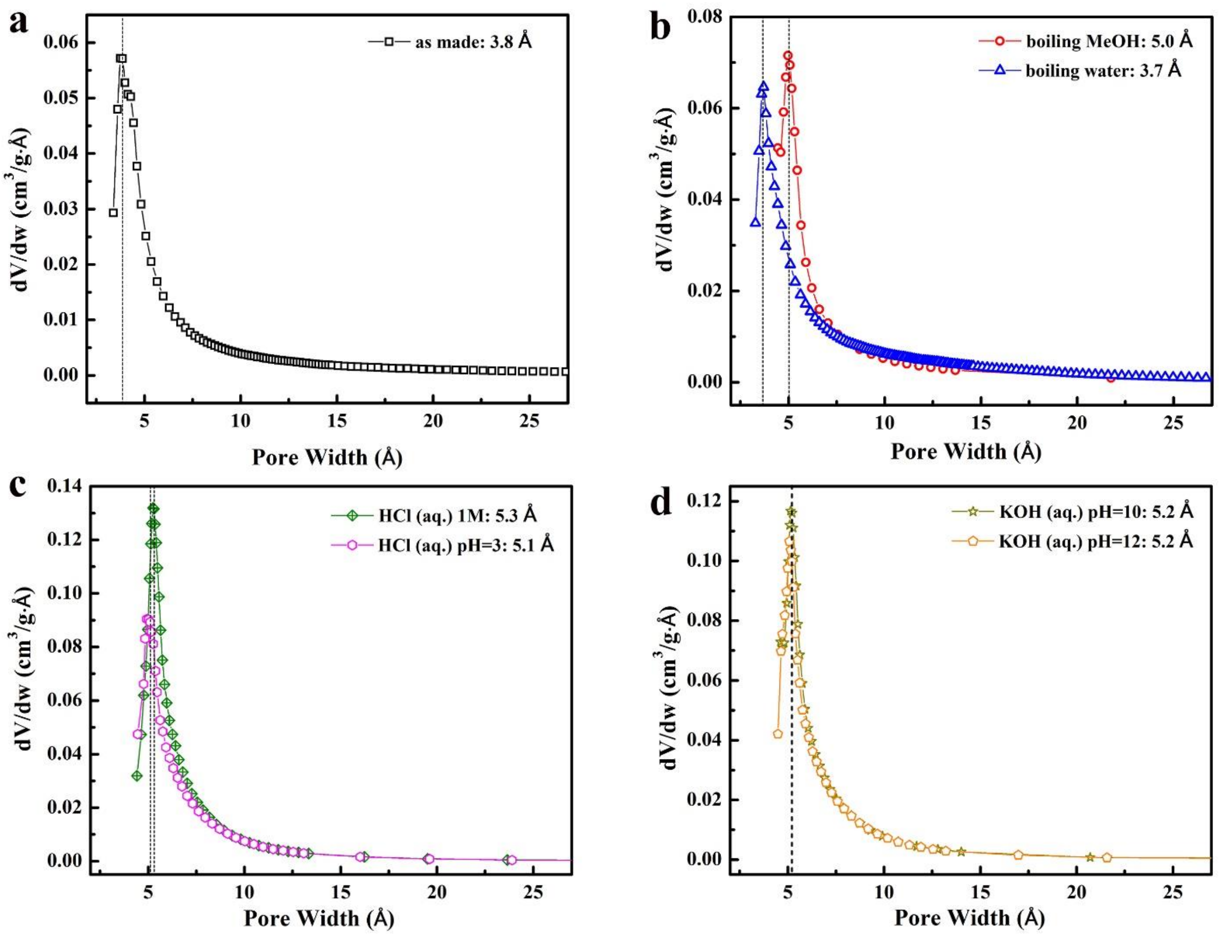

Supplementary Figure 8. Pore size distribution deduced from nitrogen adsorption isotherms for MIP-202(Zr) samples before and after various chemical treatments.

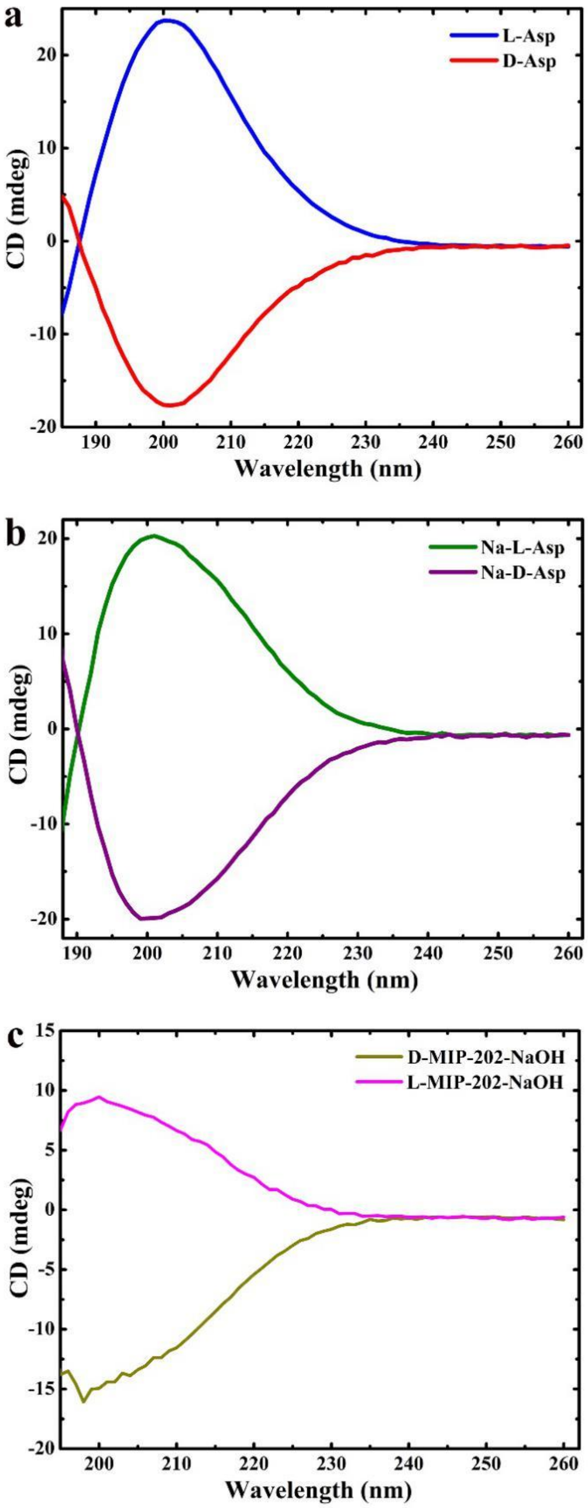

Supplementary Figure 9. Circular dichroism (CD) spectra of (a) pure L- and D-aspartic acid in aqueous solution; (b) sodium salts of pure L- and D-aspartate in aqueous solution; (c) Aqueous solution of MIP-202(Zr) samples digested in NaOH solution.

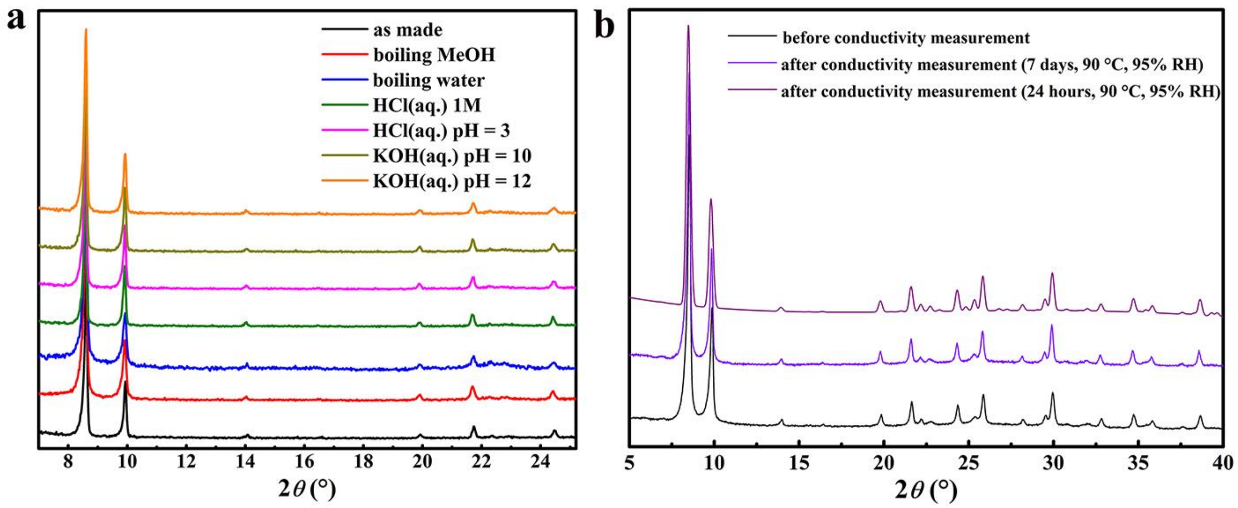

Supplementary Figure 10. PXRD pattern comparison of MIP-202(Zr) samples (a) before and after various chemical treatments; (b) before and after proton conductivity measurement for different durations.

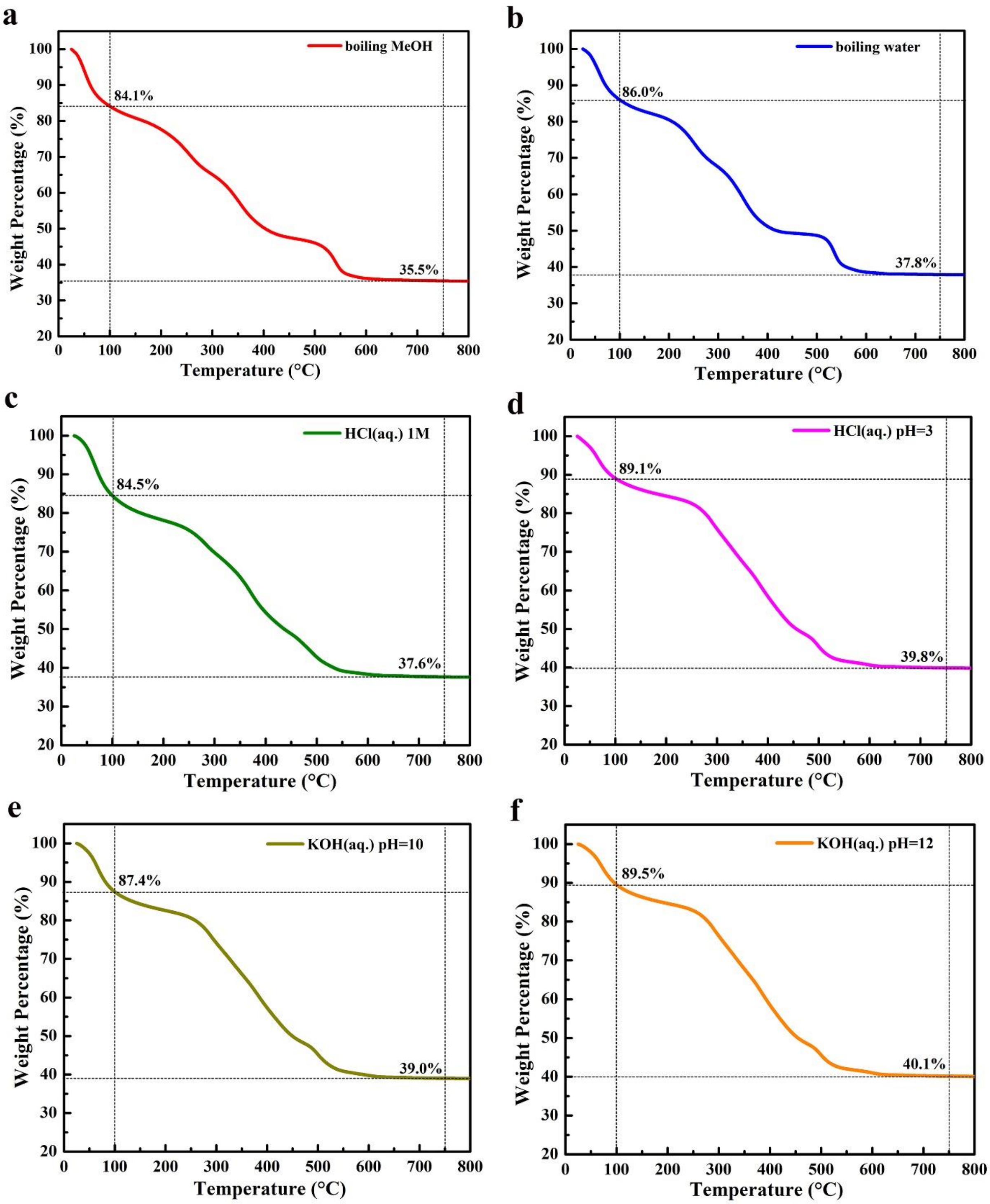

Supplementary Figure 11. TGA comparison of MIP-202(Zr) samples after various chemical treatments.

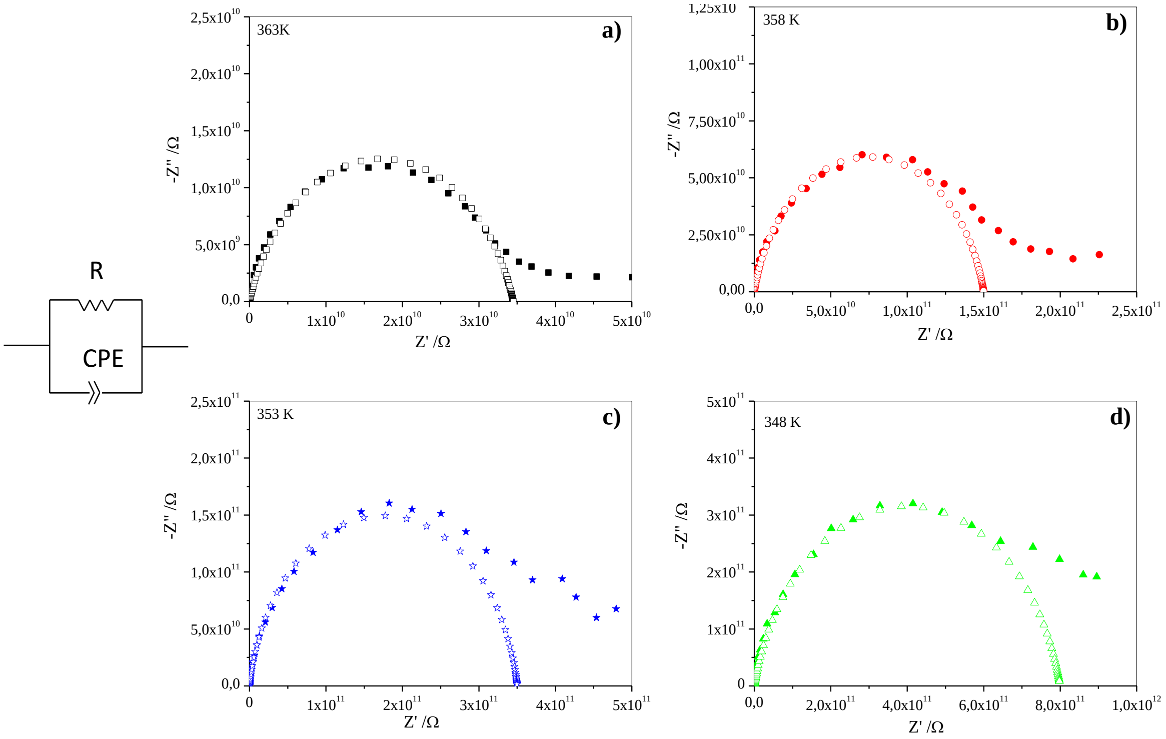

Supplementary Figure 12. Nyquist plots of the impedance of the anhydrous MIP-202(Zr) measured at RH = 0% and T = **a)** 363 K **b)** 358 K **c)** 353 K and **d)** 348 K. The filled symbols are the measured impedance data and the empty symbols correspond to the fits of the data using the equivalent circuit model detailed on the left.

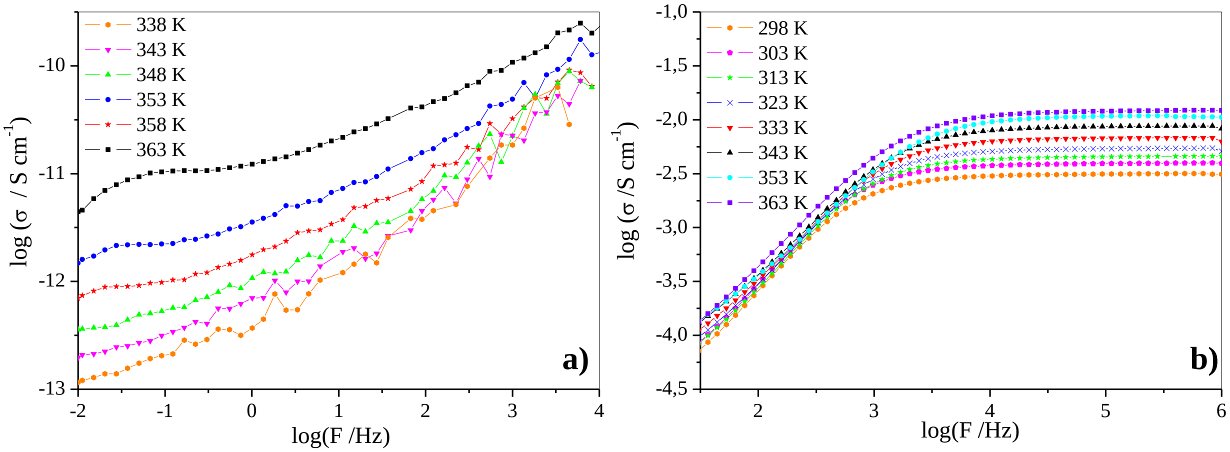

Supplementary Figure 13. Bode representation of the conductivity versus the frequency in the logarithm scale for (a) the anhydrous and (b) the hydrated MIP-202(Zr).

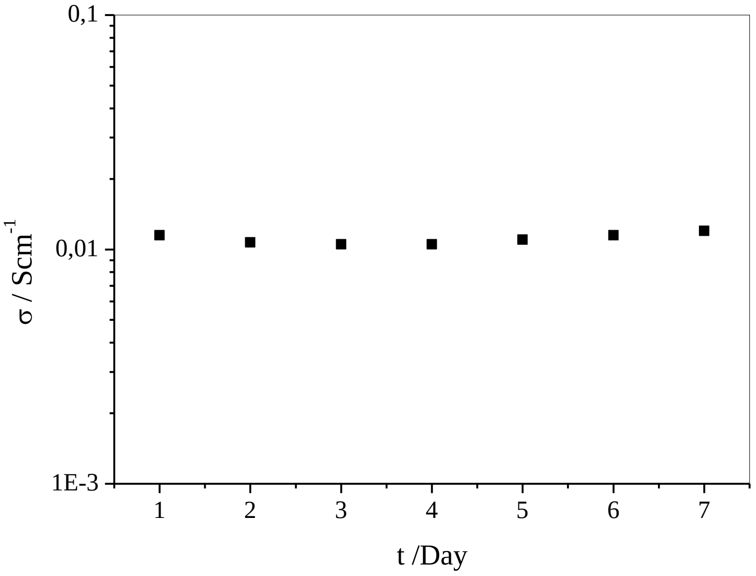

Supplementary Figure 14. Conductivity of MIP-202(Zr) recorded at 363 K and 95% RH over 7 days.

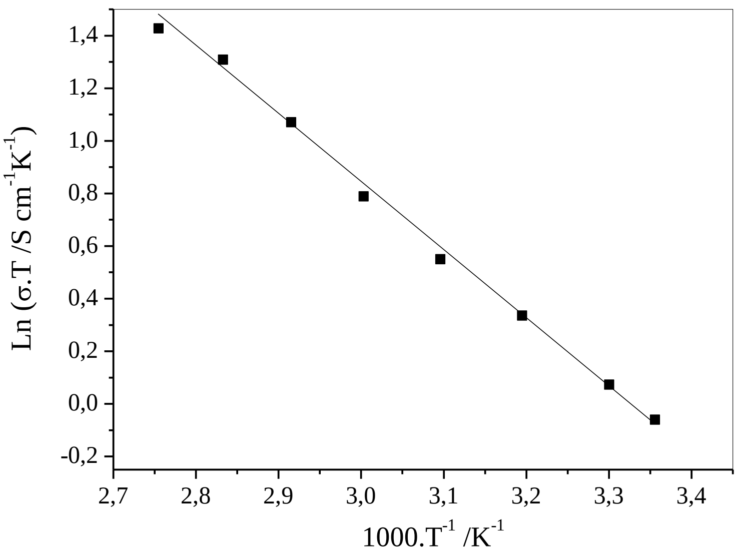

Supplementary Figure 15. Corresponding Arrhenius plot of the conductivity recorded at 95 % RH for the MIP-202(Zr). The line corresponds to the linear least-square fit.

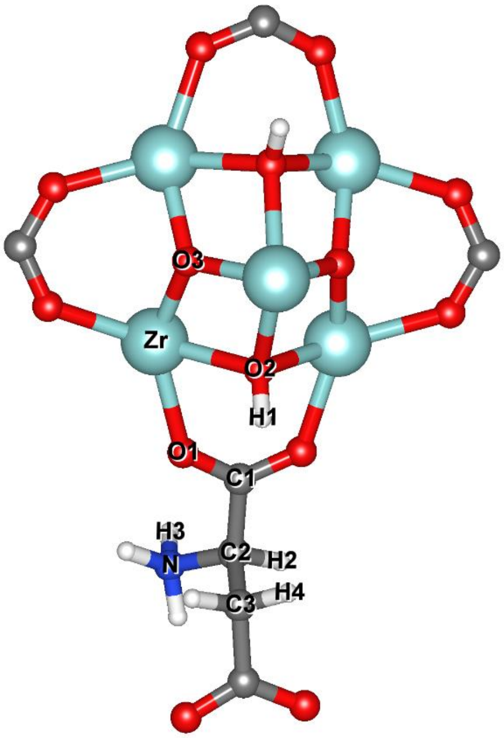

Supplementary Figure 16 Cluster model represents the atom types in MIP-202(Zr), for which the atomic partial charges are deduced and employed in the MC simulations.

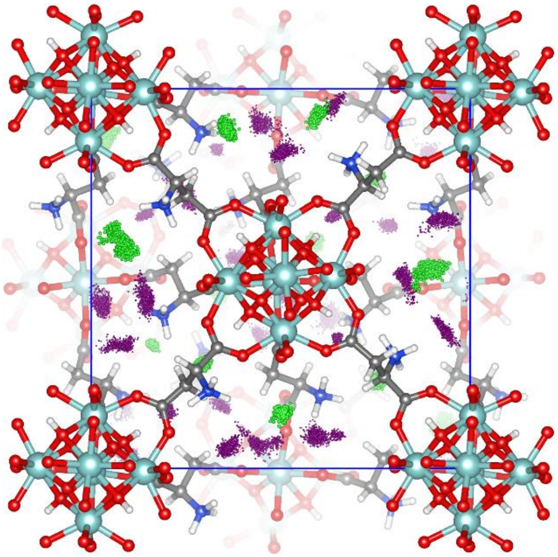

Supplementary Figure 17 Distribution of the adsorbed water molecules (purple dots) and Cl^{-} ions (green dots) in MIP-202(Zr) averaged over the MC steps calculated at 363 K.

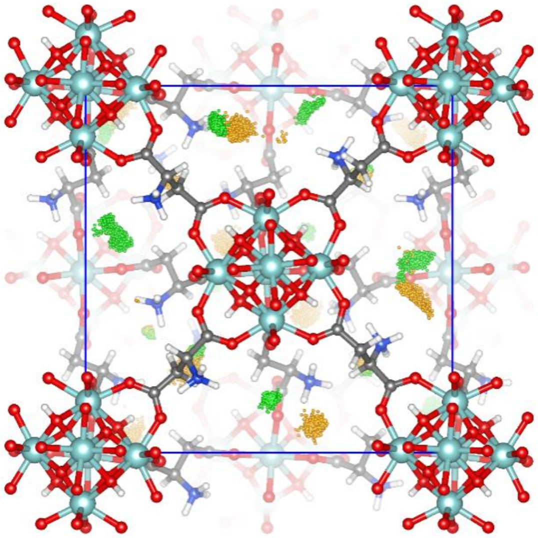

Supplementary Figure 18 Comparison of the distributions of Cl^{-} ions in anhydrous (gold dots) and hydrated phase (green dots) in MIP-202(Zr) averaged over the MC steps calculated at 363 K.

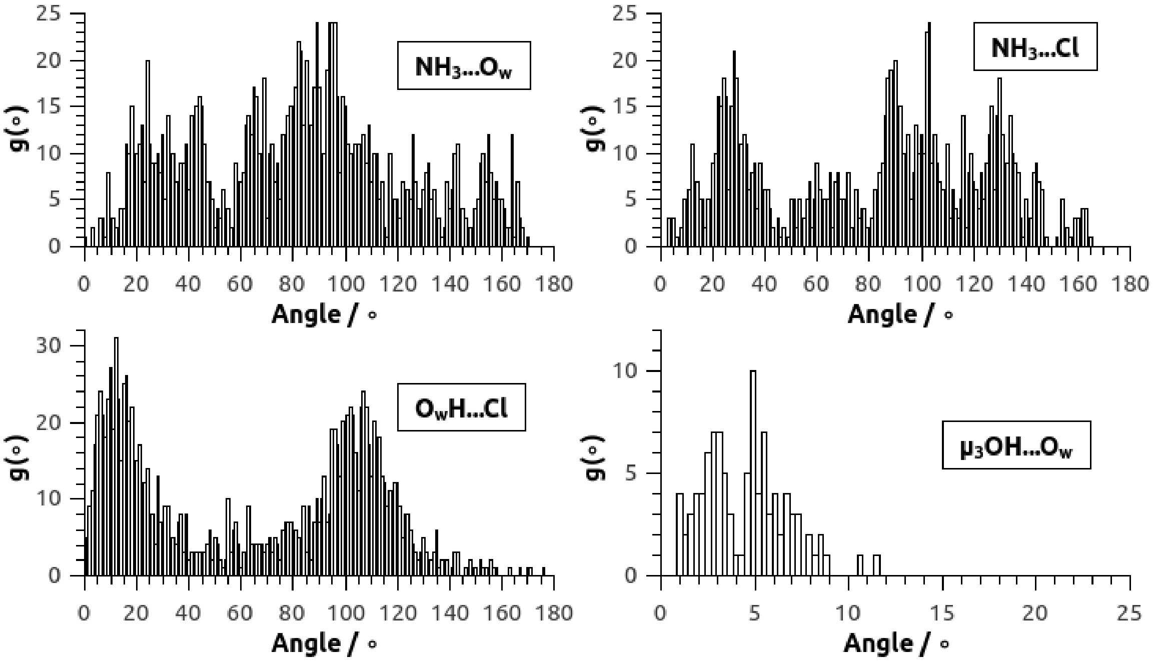

Supplementary Figure 19 Distribution of the angles between intramolecular donor-proton vector and the intermolecular donor-acceptor vector when the donor-acceptor distances are less than 3.5 Å; averaged over the MC configurations generated for the hydrated MIP-202(Zr).

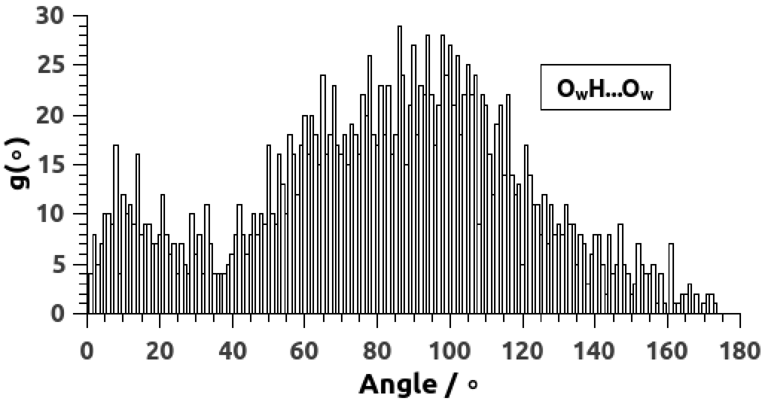

Supplementary Figure 20 Distribution of the angles between the intramolecular O-H vector and the intermolecular O-O vector when the O-O distances of the adsorbed water molecules are less than 3.5 Å; averaged over the MC configurations generated for the hydrated MIP-202(Zr).

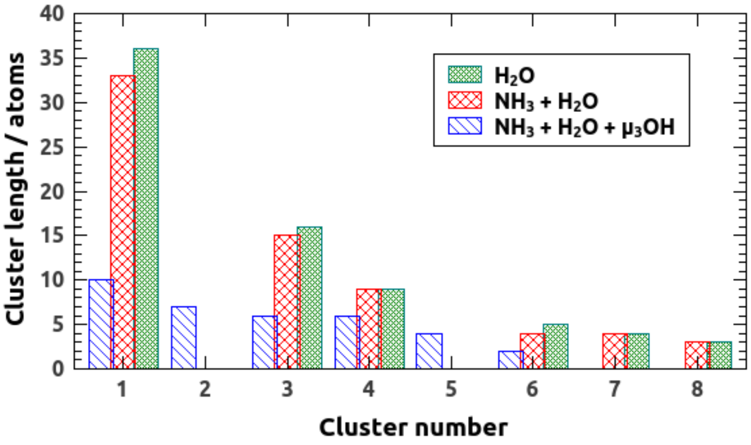

Supplementary Figure 21 Size distribution of clusters that aggregates different donor and acceptor atoms, calculated solely based on the donor-acceptor distance.

We aimed to perform quantitative analysis for the clustering of water molecules within the pores of MIP-202(Zr) based on the definition of a cluster as a continuous network of water molecules interconnected by hydrogen bonds among themselves. The criteria to define the aggregation of the water molecules in clusters were the threshold connectivity distance and the aforementioned angle between the intramolecular O-H vector and the intermolecular O-O vector. The former was defined as 3.2 to accurately represent the first interaction peak of the O-O RDF on liquid water; the latter was kept to 37°.

Supplementary Table 1. Comparison of linker availability and MOF preparation condition of reported highly proton conductive MOF (≥ 10^{-2} S cm^{-1}).

Material	Linker	Reaction Condition
**BUT-8(Cr)**	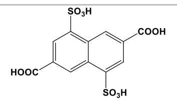 **synthetic**	solvothermal<br>190 °C/24 h
**Fe-CAT-5**	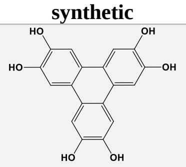 **commercial 1 g/92 € (TCI)**	solvothermal<br>180 °C/48 h
**PCMOF10**	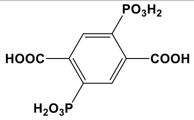 **synthetic**	solvothermal<br>150 °C/72 h
**PCMOF2½**	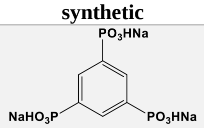 **synthetic**	solvothermal<br>120 °C/48 h
**[$(Me_2NH_2)_3(SO_4)]_2[(Zn_2(ox)_3]$**	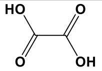 **commercial 50 g/53.1 € (Sigma)**	solvothermal<br>160 °C/96 h
**UIO-66-$(SO_3H)_2$**	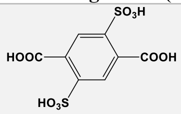 **synthetic**	1) Microwave<br>120 °C/40 min<br>2) RT oxidation
**MIL-101-SO_3H**	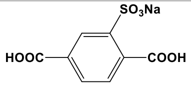 **commercial 25 g/101 € (TCI)**	solvothermal<br>190 °C/24 h
**TfOH@MIL-101**<br>**H_2SO_4@MIL-101**<br>**H_3PO_4@MIL-101**	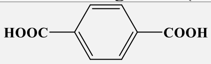 **commercial 500 g/31 € (Sigma)**	1) hydrothermal<br>220 °C/6 h<br>2) RT soaking
**H+@Ni_2(dobdc)$(H_2O)_2$**	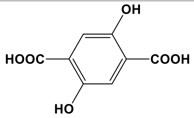 **commercial 5 g/78 € (TCI)**	1) microwave<br>110 °C/15 min<br>2) RT soaking
**MIP-202(Zr)**	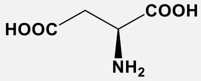 **commercial 1 kg/67 € (Fisher)**	reflux in water<br>1 h/atm.

Supplementary Table 2. SEM-EDX results of MIP-202(Zr) samples.

**Treatment**	**Cl/Zr ratio (atomic)**
**none**	54/46
**Reflux in EtOH**	51/49
**Reflux in MeOH**	51/49
**Reflux in water once**	49/51
**Soxhlet extraction with water**	31/69
**HCl 1M RT**	55/45
**HCl pH=3 RT**	50/50
**KOH pH=10 RT**	42/58
**KOH pH=12 RT**	42/58

Supplementary Table 3. Calculated formulas of MIP-202(Zr) samples after different chemical treatments from EDX and TGA results.

	**Calculated formula**	**Missing linker defect**
**Boiling MeOH**	$[Zr_6O_4(OH)_4(Aspartate)_6(HCl)_{6.24}]\cdot 18H_2O$	-
**Boiling water**	$[Zr_6O_4(OH)_4(Aspartate)_{5.76}(HCl)_{5.76}(OH)_{0.48}(H_2O)_{0.48}]\cdot 15H_2O$	4%
**HCl 1M RT**	$[Zr_6O_4(OH)_4(Aspartate)_{5.42}(HCl)_{7.33}(OH)_{1.16}(H_2O)_{1.16}]\cdot 17H_2O$	10%
**HCl pH=3 RT**	$[Zr_6O_4(OH)_4(Aspartate)_{5.59}(HCl)_6(OH)_{0.82}(H_2O)_{0.82}]\cdot 11H_2O$	7%
**KOH pH=10 RT**	$[Zr_6O_4(OH)_4(Aspartate)_{5.87}(HCl)_{4.34}(OH)_{0.26}(H_2O)_{0.26}]\cdot 13H_2O$	2%
**KOH pH=12 RT**	$[Zr_6O_4(OH)_4(Aspartate)_{5.84}(HCl)_{4.34}(OH)_{0.32}(H_2O)_{0.32}]\cdot 11H_2O$	3%

Supplementary Table 4. Elemental analysis results of MIP-202(Zr) samples after different chemical treatments.

	Calculated			Found		
	**N%**	**C%**	**H%**	**N%**	**C%**	**H%**
**Boiling MeOH**	4.16	14.28	3.78	3.61	14.74	4.20
**Boiling Water**	4.18	14.33	3.62	3.64	13.61	4.07
**HCl 1M RT**	3.79	12.99	3.79	3.07	12.05	4.30
**HCl pH=3 RT**	4.21	14.45	3.36	3.78	13.31	3.77
**KOH pH=10 RT**	4.44	15.23	3.48	3.59	13.66	4.04
**KOH pH=12 RT**	4.51	15.47	3.34	3.70	13.20	3.89

Supplementary Table 5. Conductivity of the anhydrous MIP-202(Zr) recorded at 0% RH.

T/K	Conductivity Bode representation /S cm^{-1}	Conductivity Nyquist representation /S cm^{-1}
348	3.8×10^{-13}	4.1×10^{-13}
353	9.1×10^{-13}	9.5×10^{-13}
358	2.2×10^{-12}	2.2×10^{-12}
363	1.0×10^{-11}	9.5×10^{-12}

Supplementary Table 6. Conductivity of MIP-202(Zr) recorded at 95% RH.

T/K	Conductivity Bode representation /S cm^{-1}	Conductivity Nyquist representation /S cm^{-1}
298	2.9×10^{-3}	2.8×10^{-3}
303	3.6×10^{-3}	3.2×10^{-3}
313	4.5×10^{-3}	4.0×10^{-3}
323	5.4×10^{-3}	4.7×10^{-3}
333	6.6×10^{-3}	6.0×10^{-3}
343	8.5×10^{-3}	7.5×10^{-3}
353	1.0×10^{-2}	1.0×10^{-2}
363	1.1×10^{-2}	1.1×10^{-2}

Supplementary Table 7 Atomic partial charges of MIP-202(Zr) atom types.

Atom type	Charge (e)
**H1**	0.365
**H2**	0.205
**H3**	0.300
**H4**	0.183
**C1**	0.574
**C2**	-0.158
**C3**	-0.285
**N**	-0.295
**O1**	-0.552
**O2**	-0.798
**O3**	-1.031
**Cl**	-0.700
**Zr**	2.003

Supplementary Table 8 LJ potential parameters for the atoms of MIP-202(Zr).

Atom type	σ (Å)	ε/k_B (K)
Zr	2.783	34.722
O	3.118	30.193
C	3.473	47.857
N	3.263	38.949
Cl	3.516	114.232
H	2.846	7.649

Supplementary Table 9. LJ Potential parameters and partial charges for the adsorbate molecules

Atom type	σ (Å)	ε/k_B (K)	q (e)
O_e	3.1589	93.200	0.0000
H_e	0.00	0.000	0.5564
M_e	0.00	0.000	-1.1128
N2_N	3.310	36.000	-0.4820
N2_COM	0.00	0.000	0.9640

Supplementary Note 1

Structure solution

The crystal structure of MIP-202 was solved using our newly developed software based on the AASBU strategy we described elsewhere. The geometry optimizations at the DFT level were performed using the Quickstep module[1] of the CP2K program[2,3] employing the Gaussian Plane Wave (GPW) formalism. The general gradient approximation (GGA) to the exchange-correlation functional according to Perdew-Burke-Ernzerhof (PBE)[4] was used in combination of Grimme's DFT-D3 semi-empirical dispersion corrections.[5,6] Triple-ζ plus valence polarized Gaussian-type basis sets (TZVP-MOLOPT) were considered for all atoms, except for the Zr metal centers, where double-ζ plus valence polarization functions (DZVP-MOLOPT) were employed.[7] The interactions between core electrons and valence shells of the atoms were described by the pseudopotentials derived by Goedecker, Teter, and Hutter (GTH).[8-10] The auxiliary plane wave basis sets were truncated at 400 Ry.

Monte Carlo simulations

The MC calculations were performed in the NVT ensemble at 90 °C with a simulation box of 8 conventional unit cells (2 × 2 × 2) containing 24 Cl^- and loaded with 51 H_2O molecules per unit cell as determined by the thermogravimetric analysis. The interactions between the guest water molecules and the MOF structure were described by a combination of site-to-site Lennard-Jones (LJ) contributions and Coulombic terms. A mixed set of universal force field (UFF)[11] and DREIDING force field[12] parameters were adopted to describe the LJ parameters for the atoms in the inorganic and organic part of the framework. The water molecules were described by the TIP4P/2005 potential model[13] corresponding to a microscopic representation of four LJ sites. Following the treatment adopted in other well-known force fields,[14,15] the hydrogen atoms of the

hydroxyl (μ_3OH group of inorganic nodes) and NH_3 functional groups (in the aspartic acid ligands) interact with the adsorbate water molecules only via the Coulombic potential, consistent with the strategy we validated in previous studies.[16,17] Short-range dispersion forces were truncated at a cutoff radius of 12 Å while the interactions between unlike force field centers were treated by means of the Lorentz-Berthelot combination rule. The long-range electrostatic interactions were handled using the Ewald summation technique. Hence, 2×10^8 Monte Carlo steps have been used for both equilibration and production runs. These MC calculations were performed using the Complex Adsorption and Diffusion Simulation Suite (CADSS) code.[18] To gain insight into the arrangement of the water guests in MIP-202, the guest−guest and guest−host radial distribution functions (RDFs), the density probability distributions for all of the guests and Cl^- ions, and the number of hydrogen bonds, size of the water clusters were calculated by averaging over hundreds of configurations generated during the MC simulations.

Atomic partial charge calculations

Single point energy calculations and Mulliken population analysis[19] were performed to extract the partial charges for each atom type of the MIP-202(Zr) framework using DMol3 code. These calculations were based on the PBE functional and the double numerical basis set containing polarization functions (DNP).[20]

Pore size distribution calculations

The methodology reported by Gelb and Gubbins[21] was used to calculate the pore size distributions (PSD) of the MIP-202 structures, as shown in Supplementary Figure 6. In these calculations, the van deer waals parameters of the framework atoms were adopted from the DREIDING[12] force field except for Zr atom, for which the parameters were taken from the niversal force field (UFF)[11] as they are not available in the former.

Supplementary Note 2

The data collection and refinement parameters of MIP-202(Zr) single crystal structure are indicated as following:

Crystal data

$C_{24}H_{42}N_6O_{32}Zr_6$	Dx = 1.728 g.cm^{-3}
Mr = 1473.95 g.mol^{-1}	Synchrotron radiation, λ = 0.70846 Å
Cubic, *Pn*-3	μ = 1.13 mm^{-1}
a = 17.826 (2) Å	T = 100 K
V = 5665 (2) Å^3	Needle, colorless
Z = 4	0.03 × 0.01 × 0.01 mm
F(000) = 2896	

Data collection

PROXIMA 2A - Synchrotron SOLEIL	1509 reflections with $I > 2\sigma(I)$
diffractometer	Rint = 0.170
Radiation source: synchrotron	θmax = 24.7°, θmin = 1.6°
φ scan	$h = -20 \rightarrow 19$
36542 measured reflections	$k = -20 \rightarrow 20$
1627 independent reflections	$l = -20 \rightarrow 20$

Refinement

Refinement on F^2	Hydrogen site location: inferred from
Least-squares matrix: full	neighbouring sites
$R[F^2 > 2\sigma(F^2)] = 0.140$	H-atom parameters constrained
$wR(F^2) = 0.352$	$w = 1/[\sigma^2(F_o^2) + (0.1172P)^2 + 197.4568P]$
S = 1.15	where $P = (Fo^2 + 2Fc^2)/3$
1627 reflections	(Δ/σ)max = 0.007
91 parameters	$\Delta\rho$max = 2.63 e.Å^{-3}
23 restraints	$\Delta\rho$min = −4.17 e.Å^{-3}

Supplementary Note 3

Combined analysis of EDX, TGA and elemental analysis results

In order to calculate the possible amount of structural defect generation and the corresponding structure formulas of the samples after different chemical treatments, a combined analysis of EDX, TGA and elemental analysis results was carried out as below:

The ideal formula for the MIP-202(Zr) is $[Zr_6O_4(OH)_4(Aspartate)_6(HCl)_6]{\cdot}nH_2O$. There are three possibilities of structural defects generated: 1) missing linker; 2) missing inorganic SBU and 3) both organic and inorganic moieties are missing. TGA data support that the organic parts are always less or equal to the ideal ratio for all the samples involved, which excludes the second and the third possibilities of defects. In this case, only missing linker defect will be taken into consideration for calculation. When one aspartate linker is missing, there must be two pairs of –OH/H_2O to complete the coordination vacancy and balance the charge instead. The amount of trapped HCl also could be varied along treatments under different conditions. Thus the formula of the MIP-202(Zr) sample after treatment turns to be:

$$\mathbf{[Zr_6O_4(OH)_4(Aspartate)_x(HCl)_y(OH/H_2O)_{12-2x}]{\cdot}nH_2O}$$

in which y could be calculated from EDX result while x and n could be deduced from TGA data. Then the calculated CHN percentages obtained from the resulting formula could be compared with the experimental ones in order to check the accuracy of the aforementioned calculation.

For example, the EDX result of the boiling MeOH washed sample is Cl/Zr=51/49 (atomic ratio), thus y=6.24 in this case (Supplementary Table 2). In the corresponding TGA curve (Supplementary Fig. 11a), a weight loss of 15.9% before 100 °C was ascribed to the removal of guest water molecules. The final weight percentage of 35.5% at 800 °C corresponds to the ZrO_2

residue after burning the MOF sample in oxygen. Thus a weight loss of 48.6% was signed to the decomposition of the MOF and releasing the organic part. Thereby, the sample formula was calculated to be $[Zr_6O_4(OH)_4(Aspartate)_6(HCl)_{6.24}]\cdot18H_2O$. The calculated CHN percentages from this formula are C 14.28%, H 3.78% and N 4.16%, in a good agreement with the experimental data (C 14.74%, H 4.20% and N 3.61%).

The same calculation was carried out for all the samples tested and their corresponding formulas are listed in Supplementary Table 3. In general, the calculated CHN percentages are in an acceptable error range in comparison with the experimental ones, suggesting that those calculated formulas are reasonable. Refluxing the pristine MIP-202(Zr) sample in water generates 4% of missing linker defect. Acid treatments result in increased amount of missing linker defect. Weak bases play an important role in removing trapped HCl molecules rather than making missing linker defect. Therefore, the presence of structural defect and removal of trapped HCl could be responsible for the increased uptakes in their corresponding nitrogen adsorption isotherms when compared with that of the pristine sample.

Supplementary Note 4

AC impedance measurements

Bode and Nyquist representations

In the Bode diagram, the real part of the measured *ac* conductivity ($\sigma_{ac}(\omega,T)$) typically results from the superposition of three contributions: the Maxwell Wagner Sillars response $\sigma_{MWS}(\omega,T)$, the diffusion conductivity $\sigma_{dc}(T)$ and the polarization conductivity $\sigma_{pol}(\omega,T)$ (**Equation 1**):

$$\sigma_{ac}(\omega,T) = \sigma_{MWS}(\omega,T) + \sigma_{dc}(T) + \sigma_{pol}(\omega,T), \quad \textbf{(1)}$$

where ω is the electrical field angular frequency and T is the temperature. The diffusion conductivity corresponds to long-range redistribution of charges, i.e. ionic or electron transport, while the polarization contribution arises from local rearrangement of charges or dipoles causing dipolar reorientation and thus resulting in the intrinsic bulk polarization. Maxwell Wagner Sillars (MWS) polarization is due to the accumulation of charges at the sample/electrodes interface and also depends on extrinsic parameters, such as the sample shape.

The Bode plots of the anhydrous and hydrated MIP-202(Zr) recorded at 363 K are illustrated in Supplementary Figs. 13a and 13b, respectively. The conductivity profiles of both solids are in sharp contrast, evidencing the water-mediated proton conductor behavior of MIP-202(Zr). The anhydrous material response is mainly dominated by the conductivity increase with frequency corresponding to the polarization contribution, associated with the local rearrangements of NH_3^+ and/or Cl^- species. The low frequency conductivity plateau due to the long-range displacement of charges is only observable for the higher temperature range, with values corresponding to that of an insulator ($\sigma < 10^{-11}$ $S.cm^{-1}$, Supplementary Table 5). The signal decrease at very low frequency illustrates the Maxwell Wagner Sillars response due to the accumulation of charges at the

sample/electrodes interface and consequently indicates the ionic features of the conductivity. Being opposite, the conductivity recorded at 95% RH is drastically different: the apparent conductivity plateau is centered around 10^{-3}-10^{-2} $S.cm^{-1}$ (Supplementary Table 6) and is accompanied by the Maxwell Wagner Sillars response responsible for the conductivity falling at lower frequency, whereas the polarization contribution is no more observable in the so-studied frequency range.

For comparison, the Nyquist plot of the anhydrous and hydrated MIP-202(Zr) were also considered. The bulk resistance was deduced directly from the impedance plot extrapolation in the case of the hydrated sample or from the fitting of the Nyquist plot using equivalent circuit models for the anhydrous material (Supplementary Fig. 12). Conductivity ($\sigma/S.cm^{-1}$) was calculated considering $\sigma = 1/R \times l/S$, where l and S are the sample thickness (cm) and surface (cm^2), respectively, and R is the bulk resistance of the sample (Ω).

Conductivity values deduced from the Bode diagram and the Nyquist representation are comparable (Supplementary Tables 5 and 6), supporting that both methods can be equally considered.

References

1 J. VandeVondele, M. Krack, F. Mohamed, M. Parrinello, T. Chassaing and J. Hutter, *Comput. Phys. Commun.*, 2005, **167**, 103–128.

2 J. Hutter, M. Iannuzzi, F. Schiffmann and J. VandeVondele, *Wiley Interdiscip. Rev. Comput. Mol. Sci.*, 2014, **4**, 15–25.

3 The CP2K developers group, http://www.cp2k.org.

4 J. P. Perdew, K. Burke and M. Ernzerhof, *Phys. Rev. Lett.*, 1996, **77**, 3865–3868.

5 S. Grimme, J. Antony, S. Ehrlich and H. Krieg, *J. Chem. Phys.*, 2010, **132**, 154104.

6 S. Grimme, *J. Comput. Chem.*, 2004, **25**, 1463–73.

7 J. VandeVondele and J. Hutter, *J. Chem. Phys.*, 2007, **127**, 114105.

8 S. Goedecker, M. Teter and J. Hutter, *Phys. Rev. B*, 1996, **54**, 1703–1710.

9 M. Krack, *Theor. Chem. Acc.*, 2005, **114**, 145–152.

10 C. Hartwigsen, S. Goedecker and J. Hutter, *Phys. Rev. B*, 1998, **58**, 3641–3662.

11 a. K. Rappe, C. J. Casewit, K. S. Colwell, W. a. Goddard and W. M. Skiff, *J. Am. Chem. Soc.*, 1992, **114**, 10024–10035.

12 S. L. Mayo, B. D. Olafson and W. A. Goddard, *J. Phys. Chem.*, 1990, **94**, 8897–8909.

13 J. L. F. Abascal and C. Vega, *J. Chem. Phys.*, 2005, **123**, 234505.

14 C. D. Wick, M. G. Martin and J. I. Siepmann, *J. Phys. Chem. B*, 2000, **104**, 8008–8016.

15 W. L. Jorgensen, D. S. Maxwell and J. Tirado-Rives, *J. Am. Chem. Soc.*, 1996, **118**, 11225–11236.

16 Q. Yang, S. Vaesen, F. Ragon, A. D. Wiersum, D. Wu, A. Lago, T. Devic, C. Martineau, F.

Taulelle, P. L. Llewellyn, H. Jobic, C. Zhong, C. Serre, G. De Weireld and G. Maurin, *Angew. Chemie Int. Ed.*, 2013, **52**, 10316–10320.

17 A. Cadiau, J. S. Lee, D. Damasceno Borges, P. Fabry, T. Devic, M. T. Wharmby, C. Martineau, D. Foucher, F. Taulelle, C.-H. Jun, Y. K. Hwang, N. Stock, M. F. De Lange, F. Kapteijn, J. Gascon, G. Maurin, J.-S. Chang and C. Serre, *Adv. Mater.*, 2015, **27**, 4775–4780.

18 Q. Yang and C. Zhong, *J. Phys. Chem. B*, 2006, **110**, 17776–17783.

19 R. S. Mulliken, *J. Chem. Phys.*, 1955, **23**, 1833–1840.

20 W. J. Hehre, R. Ditchfield and J. A. Pople, *J. Chem. Phys.*, 1972, **56**, 2257–2261.

21 L. D. Gelb and K. E. Gubbins, *Langmuir*, 1999, **15**, 305–308.
